# Action of Kreosote in the Treatment of Sycosis

**Published:** 1865-07

**Authors:** 


### Selections.

Action of Kreosote in the Treatment of Sycosis.—Dr. Masse called the attention of the Paris Academy of Sciences to the use of kreosote to destroy the growth of spores in parasitic affections of the skin. The idea was a deduction of Bèchamp's experiments, proving that this essential substance stops the development of spores of mucedines, and of ova of infusoria in fermentable solutions. Cryptogamous parasites resembling in their organization those growing during fermentation, Dr. M. thought that kreosote ought to act on them in the same way. The experiment was successfully tried with a young soldier having sycosis. Spores and tubes of the *mycrosporon mentagrophyte* were discovered with the microscope in the tubercles of the affected bearded portion of the face. The patient was cured after eight days of treatment, consisting in lotions, made twice daily, with fifty gram. of water, fifty alcohol, and fifty centigram. of kreosote.—*New York Med. Journal*, from *France Mèdicale.*

Dr. Richardson, an English chemist, says that iodine, placed in a small box, with a perforated lid, destroys organic poison in rooms. During the continuance of an epidemic smallpox in London he saw the method used with benefit.—*Amer. Druggists' Circular.*

